# Analysis of survival differences in advanced triple-negative breast cancer: a real-world study

**DOI:** 10.3389/fonc.2025.1635243

**Published:** 2025-08-15

**Authors:** Jun-Sheng Zheng, Xiao-Wen Wang, Zhi-Qiang Shi, Zhao Bi, Yong-Sheng Wang, Peng-Fei Qiu

**Affiliations:** Department of Breast Cancer Center, Shandong Cancer Hospital and Institute, Shandong First Medical University and Shandong Academy of Medical Sciences, Jinan, China

**Keywords:** triple-negative breast cancer, advanced breast cancer, survival, correlation, treatment

## Abstract

**Background:**

Advanced triple-negative breast cancer (aTNBC) has a poor prognosis, and there is a dearth of relevant real-world research data. This study is aimed at analyzing the survival outcomes and subgroup characteristics of aTNBC in the first-line treatment stage, providing data support for clinical treatment decisions.

**Methods:**

A retrospective analysis was conducted on 215 patients with aTNBC who received first-line salvage treatment at Shandong Cancer Hospital from January 2018 to March 2023 (74 patients of *de novo* metastatic breast cancer [dnMBC] and 141 patients of recurrent metastatic breast cancer [rMBC]). Progression-free survival (PFS) and overall survival (OS) were assessed using the Kaplan-Meier method, and hazard ratio (HR) were calculated using the Cox regression model. Spearman correlation analysis was used to evaluate the relationship between PFS and OS.

**Results:**

The median PFS for aTNBC patients during the first-line treatment phase was 8.40 months (95% CI: 7.56–9.24 months), while the median OS was 23.87 months (95% CI: 20.53–27.21 months). Multivariate Cox regression and interaction analyses identified several independent prognostic factors affecting PFS, including dnMBC, platinum-containing regimen, immunotherapy, and local treatment of metastasis. For OS, independent prognostic factors included dnMBC, G3, and platinum-containing regimen. Additional survival analysis showed that the risk of disease progression and death was significantly lower in dnMBC patients compared to rMBC patients (PFS: HR = 0.70, 95% CI: 0.51-0.95, *P* = 0.025; OS: HR = 0.65, 95% CI: 0.45-0.95, *P* = 0.023). Furthermore, in both groups, PFS and OS were positively correlated (*r*_s_ = 0.54; *r*_s_ = 0.58).

**Conclusion:**

In patients with aTNBC, those with dnMBC demonstrate a more pronounced survival benefit, with this advantage being consistent across various clinicopathological parameters. Therefore, stratifying patients by metastatic category in clinical trials may improve evaluation of treatment efficacy and support more individualized patient management.

## 1 Introduction

Breast cancer is the most common malignant tumor affecting women’s health worldwide, and its incidence and mortality rates continue to rise every year (1). Triple-negative breast cancer (TNBC) is a particularly aggressive subtype of breast cancer that lacks hormone receptors (HR) and human epidermal growth factor receptor 2 (HER-2). This unique molecular profile renders TNBC insensitive to endocrine therapy and HER-2 targeted therapy, which are effective for other breast cancer subtypes. Consequently, treatment options for TNBC are limited, and patients often face a poor prognosis (2). TNBC is characterized by its propensity for early metastasis and relatively rapid tumor growth. Studies have shown that TNBC patients are more likely to develop distant metastasis within the first few years after diagnosis compared to other breast cancer subtypes (3, 4).

The standard treatment for early-stage triple-negative breast cancer includes surgery and adjuvant chemotherapy with anthracyclines or taxanes. However, 30 to 40 percent of patients still experience recurrence or metastasis within five years, and once it occurs, the 5-year survival rate is typically less than 15% (5, 6). The primary objectives in managing advanced triple-negative breast cancer (aTNBC) are to inhibit tumor growth, prolong survival, and enhance quality of life, achieving these goals requires a deeper understanding of the clinical characteristics and biological behavior of this aggressive subtype.

Advanced breast cancer (ABC) usually encompasses two distinct clinical entities: *de novo* metastatic breast cancer (dnMBC), which is diagnosed with distant metastasis at the initial stage, and recurrent and metastatic breast cancer (rMBC), which relapses and metastasizes after early-stage tumor treatment. These two entities are classified based on the sequence of disease progression (7–9). Multiple studies have clearly indicated that there are significant differences in clinical characteristics and prognosis between patients with dnMBC and those with rMBC (10, 11). Particularly in the analysis of first-line metastatic patients, the proportion of dnMBC patients lacking secondary resistance mechanisms is higher, and this feature may have a significant impact on the research results (10). This highlights the significance of clarifying the clinical features and biological behaviors of ABC. Previous studies have indicated that in hormone receptors-positive (HR+) and human epidermal growth factor receptor 2-positive (HER-2+) ABC patients, the prognosis of dnMBC is better than that of rMBC (12–15). Moreover, the latest prospective studies have confirmed that patients with HR+/Her-2- dnMBC in the first-line treatment stage have better progression-free survival (PFS) and overall survival (OS) than those with rMBC (16).Current research mainly focuses on the clinical characteristics and treatment of aTNBC, but there are relatively few systematic comparative studies between dnMBC and rMBC, and no consensus has been reached regarding survival outcomes and prognostic factors (6, 17). Furthermore, studies have shown that in patients with aTNBC receiving first-line treatment, PFS is positively correlated with OS (18). However, it remains unclear whether this correlation also applies separately to patients with dnMBC and rMBC. Therefore, this study aims to analyze survival outcomes and prognostic factors in patients with aTNBC, with a specific focus on comparing *de novo* and recurrent metastatic breast cancer subgroups. Additionally, we evaluate whether PFS correlates with OS in each subgroup, to inform clinical treatment strategies more effectively.

## 2 Materials and methods

### 2.1 Study design

This study is a retrospective cohort study, with subjects being stage IV breast cancer patients who visited the Breast Cancer Center at the Shandong First Medical University Affiliated Cancer Hospital and received first-line treatment between January 1, 2018, and April 15, 2023. The classification of stage IV breast cancer patients is based on the TNM staging criteria of the American Joint Committee on Cancer (AJCC) (19), and they are divided into dnMBC and rMBC. dnMBC refers to cases where metastatic disease is detected within three months of the initial breast cancer diagnosis, while rMBC includes patients initially diagnosed with stage I–III breast cancer who develop metastatic recurrence at least three months after the initial diagnosis. This time frame was chosen to maintain consistency with previous studies (17). The study has been approved by the Ethics Committee of the Shandong First Medical University Affiliated Shandong Cancer Hospital, and all methods comply with the ethical guidelines of the Declaration of Helsinki. Given the retrospective nature of the data, informed consent was waived for this study.

### 2.2 Therapeutic strategies for patients with aTNBC

The selection of immunotherapy agents is primarily guided by the Combined Positive Score (CPS). A CPS score of ≥1 typically supports the recommendation for patients to receive immunotherapy in combination with chemotherapy. The choice of chemotherapy agents, such as platinum-based regimens, is mainly determined by factors including the patient’s prior treatment history, drug tolerance, and the risk or presence of adverse reactions. All patients were treated according to the established treatment plan until intolerable toxic reactions occurred or the disease progressed. The detailed treatment regimens was provided in the **Supplementary Materials** (**Supplementary Tables S3–S8**).

### 2.3 Inclusion and exclusion criteria

The inclusion criteria are as follows: (1) Female patients aged between 18 and 75 years; (2) Patients who are first diagnosed with ABC and receive first-line salvage treatment; (3) ECOG performance status (PS) of 0-1.

The exclusion criteria include: (1) HR expression greater than 1%, or HER2+; (2) Bilateral (synchronous) breast cancer; (3) Coexisting other malignant tumors and disease, such as lung cancer; (4) Incomplete patient medical records.

### 2.4 Patient data collection

The collection of patient information included age at diagnosis of ABC, body mass index (BMI), menstrual status at diagnosis of ABC, pathological classification (It is classified into invasive ductal carcinoma and other pathological classification, which are collectively labeled as “others” in the chart.), histological grade (It is based on the SBR grading system, classified as G1, G2, G3, and unknown), visceral metastasis status and number of metastases (It is divided into single-organ and multi-organ metastases, without considering the specific number of lesions within an organ). In addition, information on first-line treatment was also gathered, including immunotherapy, chemotherapy regimens, and local treatment of metastatic lesions. All enrolled patients began treatment within 7 days of confirmed recurrence or metastasis of breast cancer.

### 2.5 Study endpoints and follow-up

The study endpoints were PFS and OS. PFS is defined as the time from the initiation of first-line treatment to the first documented disease progression (based on RECIST criteria) or death from any cause, whichever occurs first. OS is defined as the time from the diagnosis of ABC to death from any cause. Patients were assessed every three months during the first year, twice a year for the following four years, and annually thereafter. Follow-up data were available until November 1, 2024. These data were primarily collected from hospital medical records and telephone interviews. In addition, information regarding adverse effects (AEs) experienced by patients during treatment will be collected from hospital medical records and telephone interview. During the follow-up process, patients were informed about the purpose of the follow-up and the research.

### 2.6 Data analysis methods

#### 2.6.1 Quantitative and qualitative data processing

Quantitative data were analyzed using t-test, while qualitative data were handled with chi-square test and Fisher’s exact test, and were described in percentage.

#### 2.6.2 Survival analysis and comparison of differences between groups

PFS and OS between the two groups were compared using Kaplan-Meier analysis and log-rank tests. The hazard ratio (HR) and 95% confidence interval (95% CI) were calculated using the Cox proportional hazards regression model, *P* < 0.05 was considered statistically significant.

#### 2.6.3 Univariate and multivariate Cox regression analysis and interaction effects

Univariate and multivariate COX regression analyses were performed to assess the clinical and pathological factors associated with survival in patients with triple-negative advanced breast cancer. To ensure a comprehensive evaluation of prognostic factors, all variables were included in the multivariate COX regression model to assess their independent effects on survival. For the variables with statistical significance (*P* < 0.05) in the multivariate analysis, interaction analysis was conducted to explore whether there were interactions among the variables.

#### 2.6.4 Correlation analysis of PFS and OS

In this study, we first performed a normality test on the PFS and OS data using the Shapiro-Wilk test to assess whether the data followed a normal distribution. If the data were normally distributed, the Pearson correlation coefficient was applied to evaluate the linear relationship between PFS and OS. If the data deviated from normality, the Spearman rank correlation coefficient was used to assess the monotonic relationship between the two.

Data analysis was performed using RStudio software and Graphical plotting was based on GraphPad Prism version 10.1.2.

## 3 Results

### 3.1 Patient characteristics

**Figure 1:**
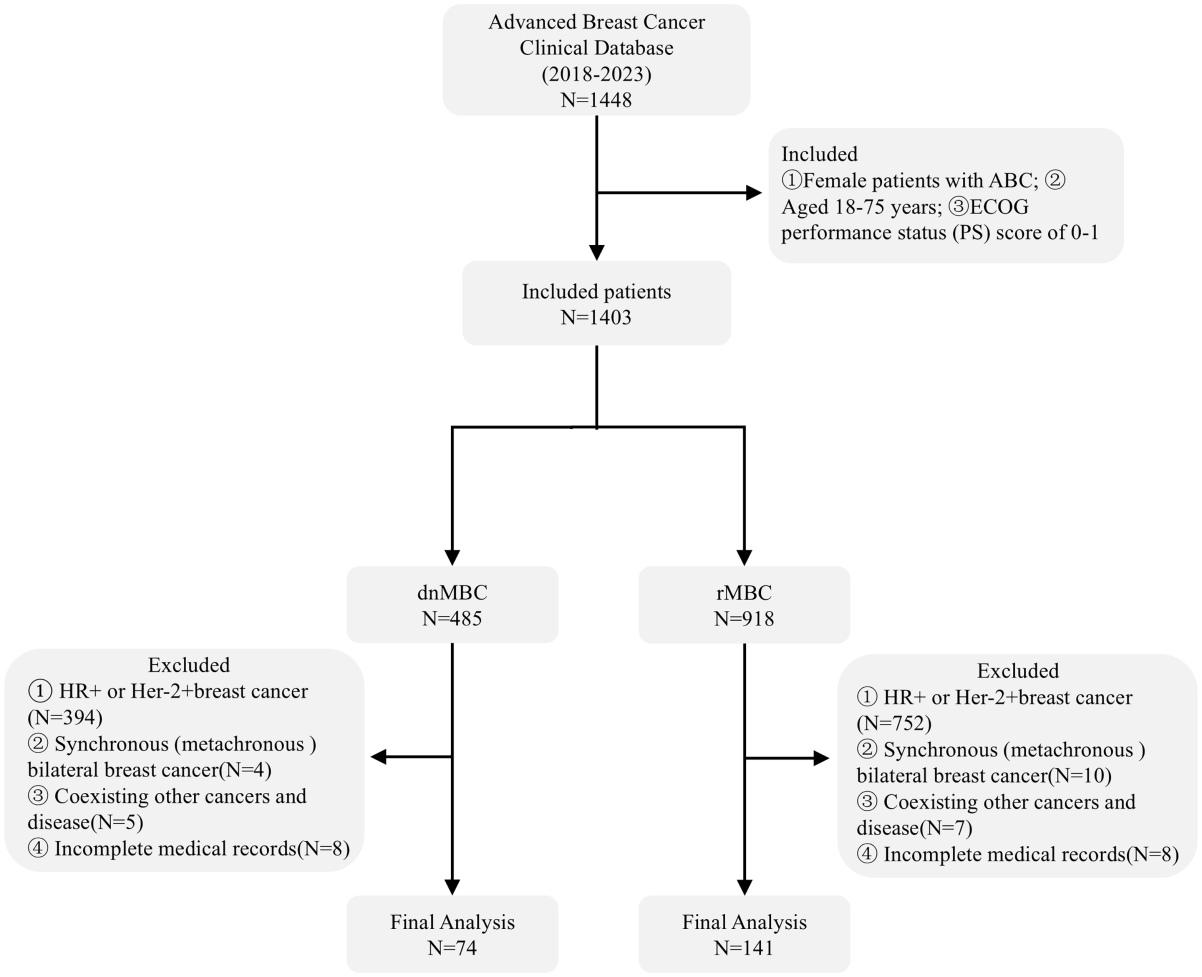
Process flowchart.

A total of 215 patients with aTNBC were included in the final analysis of this study, including 74 cases of dnMBC and 141 patients with rMBC (**Figure 1**). The median age of patients with dnMBC was 52 years old, while that of patients with rMBC was 51 years old. No significant differences were observed between the two groups regarding age at diagnosis, BMI, pathological classification, histological grade, metastatic number and whether to accept immunotherapy (*P* > 0.05) (**Table 1**).

**Table 1:** Characteristics of patient diagnosed with de novo and recurrent metastatic breast cancer.

Characteristic	All eligible(N=215) N (%)	dnMBC (N = 74) N (%)	rMBC (N = 141) N (%)	*P* value
Median age, years (range)^a^	52 (45-59)	52 (44-62)	51 (45-57)	0.926
Age at MBC diagnosis				0.138
<50	85 (39.5)	26 (35.1)	59 (41.8)	
≥50	130 (60.5)	48 (64.9)	82 (58.2)	
BMI				0.957
<18.5	16 (7.4)	6 (8.1)	10 (7.1)	
18.5-23.9	89 (41.4)	30 (40.5)	59 (41.8)	
≥24	110 (51.2)	38 (51.4)	72 (51.1)	
Menstrual state				0.349
Pre-menopause	57 (26.5)	23 (31.1)	34 (24.1)	
Post-menopause	158 (73.5)	51 (68.9)	107 (75.9)	
Histological grade^b^				0.852
G1	7 (3.2)	3 (4.1)	4 (2.8)	
G2	107 (49.8)	38 (51.4)	69 (48.9)	
G3	96 (44.7)	32 (43.2)	64 (45.4)	
Unknown	5 (2.3)	1 (1.4)	4 (2.8)	
Pathological classification				0.467
Others	36 (16.7)	10 (13.5)	26 (18.4)	
Invasive ductal carcinoma	179 (83.3)	64 (86.5)	115 (81.6)	
Viscera metastasis				0.056
No	57 (26.5)	26 (35.1)	31 (22.0)	
Yes	158 (73.5)	48 (64.9)	110 (78.0)	
Metastatic number				0.674
1	96 (44.7)	35 (47.3)	61 (43.3)	
>1	119 (55.3)	39 (52.7)	80 (56.7)	
Systemic treatment				0.373
Paclitaxel/Anthracycline-based regimen	88 (41.0)	35 (47.3)	53 (37.6)	
Platinum-based regimen	87 (40.4)	26 (35.1)	61 (43.3)	
Immunotherapy	40 (18.6)	13 (17.6)	27 (19.1)	
Local treatment of metastasis				0.066
No	65 (30.2)	58 (78.4)	92 (65.2)	
Yes	150 (59.8)	16 (21.6)	49 (34.8)	

^*^Statistically significant.

a Median age at initial diagnosis, years (Interquartile Range).

b Patients with unknown feature excluded from subgroup analysis.

dnMBC, de novo metastatic breast cancer; rMBC, recurrent metastatic breast cancer.

In the first-line treatment of dnMBC patients, the primary regimen contained platinum-based chemotherapy with carboplatin, epirubicin + cyclophosphamide followed by docetaxel. Additionally, albumin-bound paclitaxel (with or without capecitabine as intensification) and immune checkpoint inhibitor + chemotherapy were commonly used. For rMBC patients, treatment regimens were similar to those for dnMBC, but platinum-based chemotherapy with cisplatin or carboplatin was more frequently administered. Furthermore, albumin-bound paclitaxel (with or without capecitabine as intensification), capecitabine only or in combination, and immune checkpoint inhibitor + chemotherapy were also widely adopted (**Supplementary Material**, **Supplementary Figure S1**).

### 3.2 Treatment-related AEs

The most common AEs of any grade included neutropenia (78.6%), anemia (72.5%), nausea or vomiting (69.8%), peripheral neuropathy (32.1%), transaminase increasing (22.8%) (**Supplementary Material**, **Supplementary Table S9**).

### 3.3 Survival analysis of aTNBC, dnMBC, and rMBC patients

The median follow-up time of the entire cohort was 36.60 months (95% CI 32.21-40.99 months) in this study. The median follow-up time for dnMBC was 31.03 months (95% CI 20.61-41.45 months), and rMBC was 36.60 months (95% CI 34.29-38.91 months).

**Figure 2:**
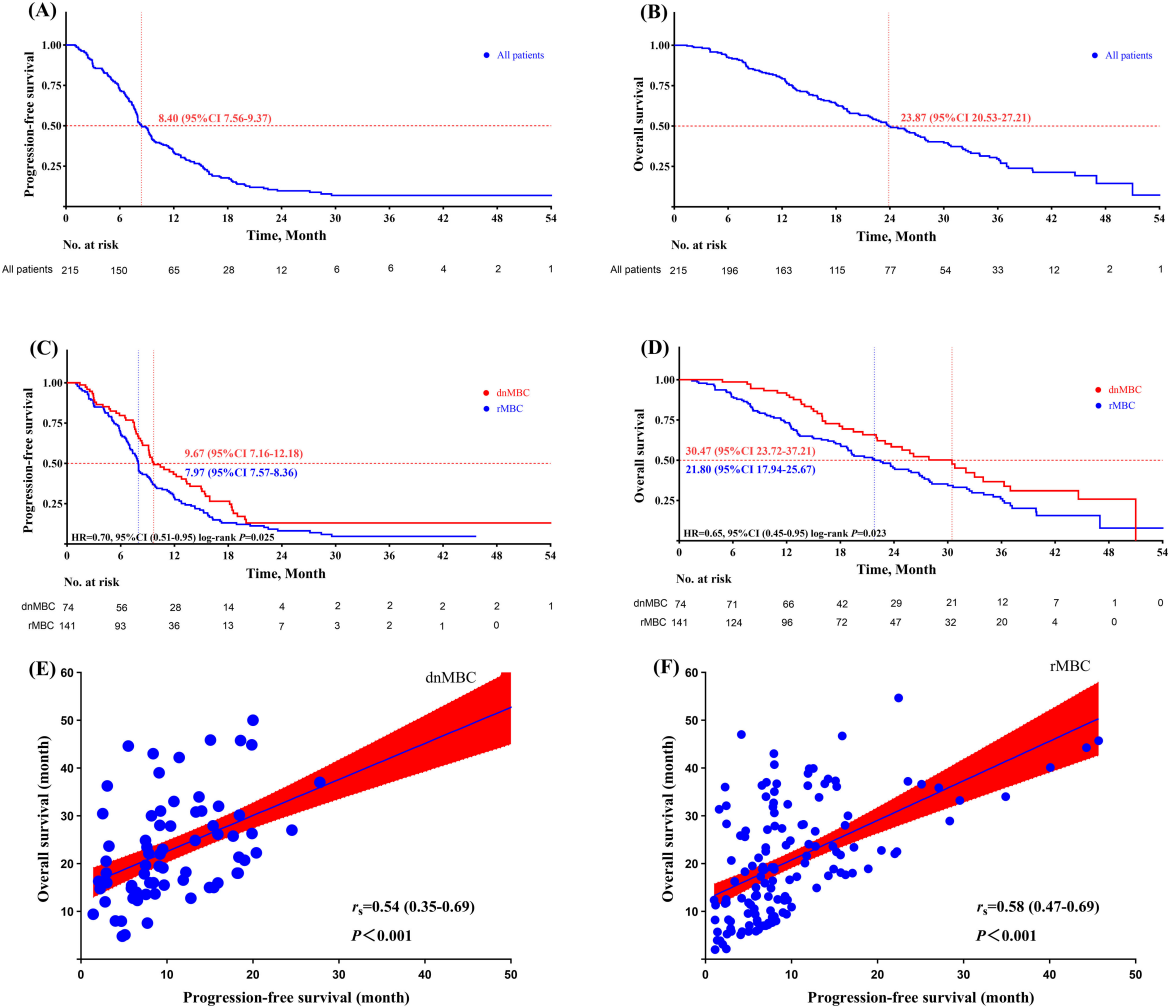
**(A)** progression-free survival of aTNBC paient; **(B)** overall survival of aTNBC paient; **(C)** progression-free survival comparison between dnMBC and rMBC palients; **(D)** overall survival comparison between dnMBC and rMBC patients; **(E)** corelation analysis of progression-free survival and overall survival within the dnMBC group; **(F)** Corelation analysis of progression-free survival and overall survival within the rMBC group.

The median PFS (mPFS) for aTNBC patients receiving first-line salvage treatment was 8.40 months (95% CI: 7.56-9.24 months), while the median OS (mOS) was 23.87 months (95% CI: 20.53-27.21 months) (**Figures 2A, B**). Patients with dnMBC receiving first-line salvage treatment exhibited a mPFS of 9.67 months (95% CI 7.16-12.17 months), whereas those with rMBC had a mPFS of 7.97 months (95% CI 7.57-8.36 months), a difference that was statistically significant (*P* = 0.025). Compared to rMBC patients, dnMBC patients had a 30% reduced risk of disease progression (HR = 0.70, 95% CI 0.51-0.95, *P* = 0.025) (**Figure 2C**). The mOS for dnMBC and rMBC patients were 30.47 months (95% CI 23.72-37.21 months) and 21.80 months (95% CI 17.94-25.66 months), respectively, with a significant difference observed between the groups (*P* = 0.023). Furthermore, dnMBC patients exhibited a 35% lower risk of disease progression compared to rMBC patients (HR = 0.65; 95% CI 0.45-0.95; *P* = 0.023) (**Figure 2D**).

Furthermore, the results of this study also revealed significant differences in survival time among different treatment regimens. Specifically, the mPFS for the paclitaxel/anthracycline regimen, platinum-based regimen, and immunotherapy regimen were 7.00 months (95% CI 5.11-8.89), 9.63 months (95% CI 8.79-9.95), and 8.43 months (95% CI 4.78-12.09), respectively. The corresponding mOS were 18.23 months (95% CI 13.11-23.37), 27.87 months (95% CI 20.07-34.66), and 22.77 months (95% CI 13.67-30.85), respectively (**Supplementary Material**, **Supplementary Figures S2**, **S3**).

### 3.4 Univariate/multivariate cox regression analysis and interaction effects for aTNBC patients

Univariate Cox regression analysis indicated that in patients with aTNBC receiving first-line salvage therapy, dnMBC (HR = 0.70, 95% CI 0.51-0.95, *P* = 0.025), platinum-containing regimen (HR = 0.72, 95% CI 0.52-0.99, *P* = 0.044), immunotherapy (HR = 0.60, 95% CI 0.40-0.92, *P* = 0.020) and local treatment of metastasis (HR = 0.75, 95% CI 0.55 - 0.96, *P* = 0.025) were all significant prognostic factors affecting PFS. The results of the multivariate regression analysis revealed that dnMBC (HR = 0.61, 95% CI 0.43-0.86, *P* = 0.004), platinum-containing regimens (HR = 0.62, 95% CI 0.44-0.89, *P* = 0.009), immunotherapy (HR = 0.46, 95% CI 0.29-0.73, *P* = 0.001), and local treatment of metastasis (HR = 0.66, 95% CI 0.46-0.96, *P* = 0.028) were all independent prognostic factors for PFS. Further interaction analysis showed no significant interactions between dnMBC and any of the aforementioned independent prognostic factors (**Supplementary Material**, **Supplementary Table S1**).

When analyzing the prognostic factors for OS, dnMBC (HR = 0.65, 95% CI 0.45-0.95, *P* = 0.023), multi-organ metastatic lesions (metastatic sites>1) (HR = 1.43, 95% CI 1.02-2.02, *P* = 0.041) and platinum-containing regimen (HR = 0.61, 95% CI 0.42-0.89, *P* = 0.011) were identified as a significant factor influencing patient survival. Notably, dnMBC (HR = 0.60, 95% CI 0.38-0.93, *P* = 0.012), G3 (HR = 4.98, 95% CI 1.46-17.04, *P* = 0.010) and platinum-containing regimen (HR = 0.72, 95% CI 0.52-0.99, *P* = 0.001) retained its status as an independent prognostic factor even after adjustment for other potential confounding factors. In addition, the interaction analysis shows that dnMBC has no mutual influence with other factors (**Supplementary Material**, **Supplementary Table S2**).

### 3.5 Correlation analysis of PFS and OS within the dnMBC and rMBC groups

Furthermore, in both the dnMBC and rMBC groups, there was a significant positive correlation between PFS and OS (dnMBC: *r*_s_ = 0.54, 95% CI 0.35-0.69, *P* <0.001; rMBC: *r*_s_ = 0.58, 95% CI 0.47-0.69, *P* <0.001). This result indicates that a longer PFS is closely associated with improved OS in both groups (**Figures 2E, F**).

### 3.6 Exploratory subgroup analysis

**Figure 3:**
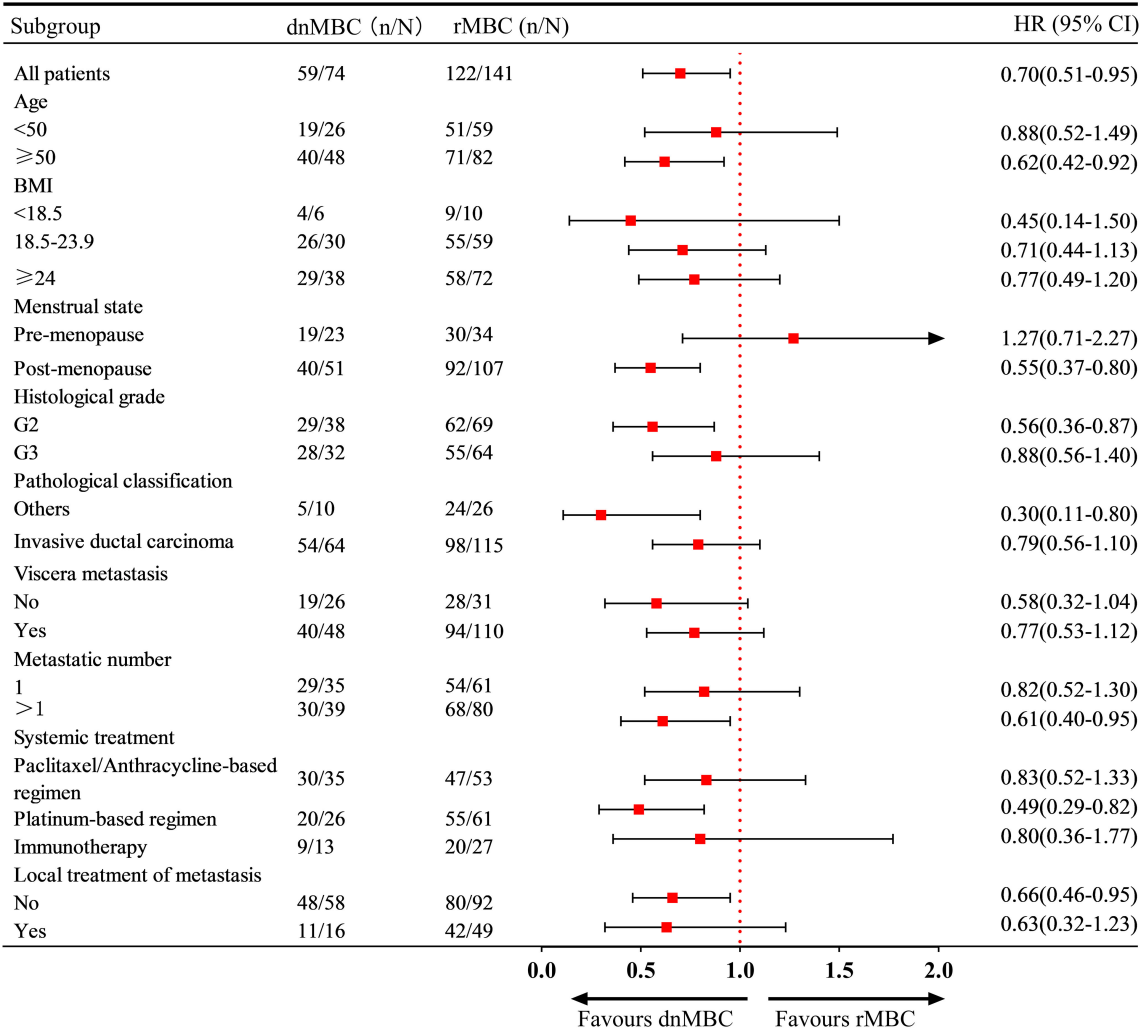
Subgroup analysis for progression-free survival.

Subgroup analysis revealed that in patients aged ≥50 years, with post-menopausal status, G2, other pathological classification and multi-organ metastases, the risk of disease progression was significantly lower in dnMBC patients than in rMBC patients. Moreover, first-line platinum-based salvage therapy was associated with improved PFS in patients in the dnMBC group (HR = 0.49, 95% CI: 0.29 - 0.82) (**Figure 3**).

**Figure 4:**
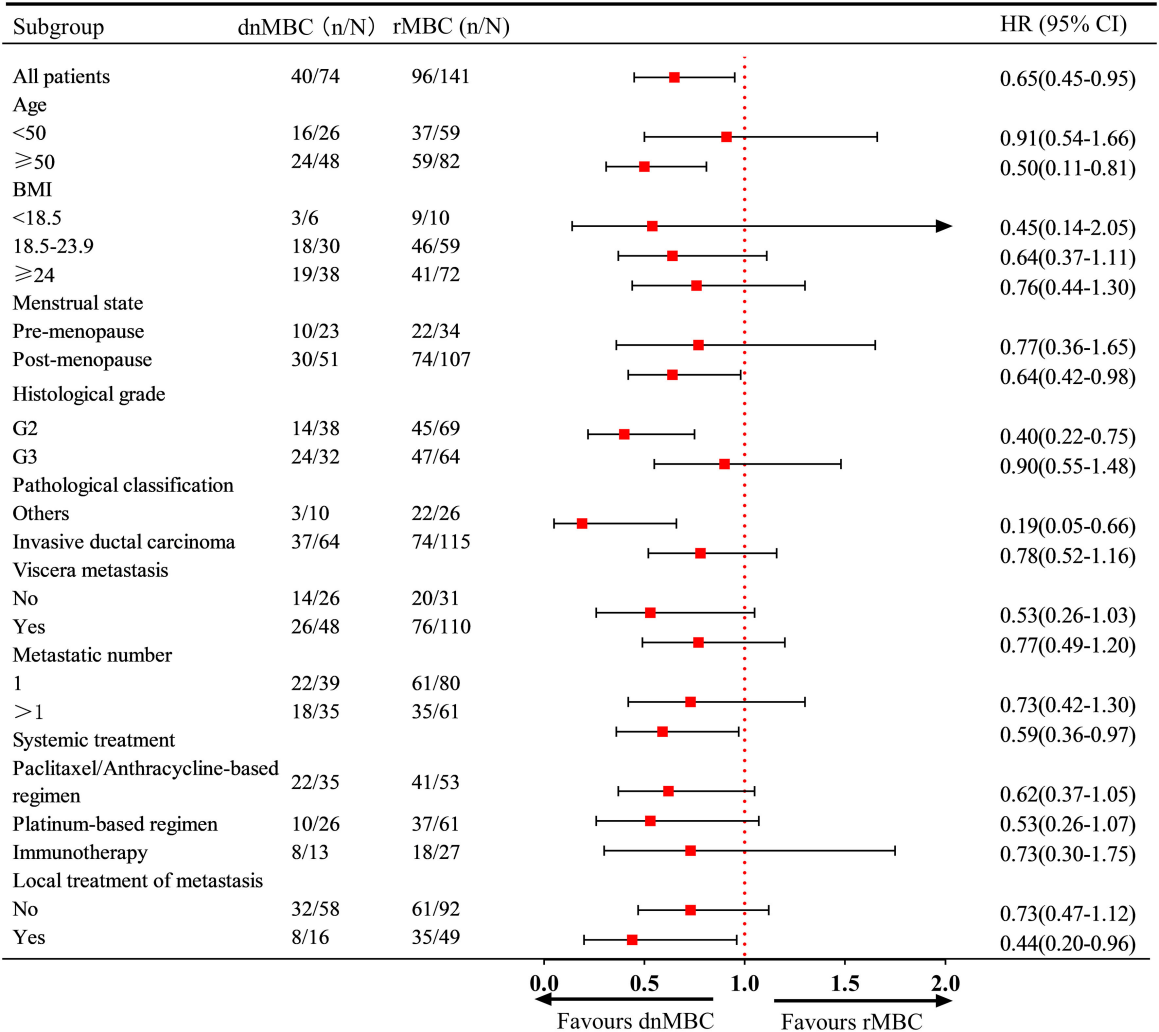
Subgroup analysis for overall survival.

Regarding the risk of death, the subgroup analysis further revealed that dnMBC patients aged ≥50 years, post-menopausal status, G2, and multi-organ metastases had a significantly lower risk of death than rMBC patients. Similarly, first-line immunotherapy was associated with improved OS in patients in the dnMBC group (HR: 0.44, 95% CI: 0.20 - 0.96) (**Figure 4**).

## 4 Discussion

In the management of aTNBC, first-line salvage therapy is a critical strategy for controlling disease progression and improving patient outcomes (18, 20). Consequently, an in-depth evaluation of the clinical characteristics of aTNBC patients, including demographic factors, tumor biology, and responses to initial treatment, is essential.

This study conducted a detailed prognosis analysis on patients with aTNBC who received first-line salvage treatment between 2018 and 2023. The results showed that the mPFS for aTNBC patients was 8.40 months (95% CI: 7.56-9.24 months), while the mOS was 23.87 months (95% CI: 20.53-27.21 months). In contrast, a real-world study in the United States, which included patient data from 2010 to 2016, reported a median OS of 11.8 months (95% CI: 10.2 - 13.1 months) and a median PFS of 4.2 months (95% CI: 3.7 - 4.6 months) (21). Additionally, a real-world study conducted in Denmark included patient data from 2017 to 2019, reporting a mPFS of 4.9 months (95% CI: 4.2 - 6.3 months) and a mOS of 11.6 months (95% CI: 9.9 - 17.3 months) (22). These comparison results indicate that in real-world settings, the prognosis of aTNBC patients has significantly improved over time, which may be attributed to a deeper understanding of aTNBC and advancements in anti-tumor treatments (6). Multivariate COX regression analysis revealed that age, BMI, menstrual status, pathological classification, and visceral metastasis status were not independent prognostic factors for PFS and OS. However, multivariate COX regression analysis demonstrated that disease status (dnMBC or rMBC) was an independent prognostic factor affecting both PFS and OS of patients, indicating that dnMBC and rMBC represent distinct patient groups with different prognoses. The survival analysis results of our study indicated that the PFS and OS of patients with dnMBC were significantly better than those of patients with rMBC (mPFS: 9.67 vs. 7.97 months, *P* = 0.025; mOS: 30.47 vs. 21.80 months, *P* = 0.023). However, File et al.’s research found that among patients with aTNBC receiving first-line salvage therapy, there was no statistically significant difference in mPFS and mOS between patients with dnMBC and those with rMBC. Nevertheless, the mPFS and mOS of dnMBC patients were still better than those of rMBC patients (mPFS: 4.00 vs. 3.00 months, *P* = 0.121; mOS: 19.20 vs 13.80 months, *P* = 0.115) (17). We speculate that patients with dnMBC, having not received any anti-tumor treatment, are less likely to develop drug resistance (14, 23). In contrast, patients with rMBC who have undergone neoadjuvant or adjuvant therapy may be more prone to drug resistance, and their tumor heterogeneity is more complex, which leads to a poorer prognosis (24, 25). The study by Lobbezoo et al. revealed that, after excluding patients who received (neo)adjuvant systemic therapy, the prognosis of patients with dnMBC was significantly better than that of patients with rMBC, specifically those with a disease-free interval (DFI) of less than 24 months (adjusted HR = 1.69, 95% CI 1.11 - 2.58) (26). However, this study did not focus on aTNBC. Furthermore, Garrido-Castro et al. conducted targeted exon sequencing on 929 patients with ABC and found that CIITA mutations were more prevalent in triple-negative dnMBC patients compared to rMBC patients (27). These mutations may enhance immune escape by reducing MHC II expression and promoting MYB amplification. Immune escape is generally associated with poorer survival outcomes (28). In addition, the study further revealed that patients with dnMBC who exhibited a high tumor mutational burden (TMB) demonstrated significantly improved OS (*P* = 0.041), suggesting that elevated TMB may enhance tumor immunogenicity and improve responsiveness to immunotherapy. In contrast, no significant association between TMB and OS was observed in rMBC (*P* = 0.350), indicating that the prognostic significance of TMB may vary across different subtypes of breast cancer. However, the results from the exon sequencing complicate the explanation for the differences between dnMBC and rMBC. Notably, the survival data from this study showed no significant difference in overall survival between dnMBC and rMBC patients in the triple-negative breast cancer subgroup (20.0 vs 22.5 months, *P* = 0.79). This finding suggests that although genomic features reveal potential biological differences, they do not translate into significant survival prognostic differences. Therefore, further research is needed to explore whether there are differences between these two groups of patients and the reasons for such differences.

Additionally, the results indicate that PFS is positively correlated with OS in both the dnMBC and rMBC cohorts, consistent with the findings of Courtinard et al. (*r* = 0.73-0.81) (18). And, The study by Cabel et al. study revealed that the PFS of first-line treatment is an independent prognostic factor for the OS of patients receiving third-line or fourth-line treatment. This finding further emphasizes the importance of PFS in first-line treatment in clinical practice. Subgroup analysis also revealed that patients with dnMBC had a lower risk of disease progression and death compared to those with rMBC in specific subgroups such as aged ≥ 50, post-menopausal status, G2, other pathological classification and multi-organ metastases. It is noteworthy that, except for the premenopausal subgroup, the dnMBC group showed a trend toward lower risks of disease progression and death compared to the rMBC group in all other subgroups. In the subgroup of patients aged < 50, no significant differences in progression or mortality risks were observed between the dnMBC and rMBC groups. However, in the subgroup aged ≥ 50, the dnMBC group demonstrated significantly improved PFS and OS compared to the rMBC group. These findings suggest that in the ≥ 50 age group, the poorer PFS and OS outcomes in the rMBC group may be attributed to longer disease duration and the development of treatment resistance over time. These subgroup characteristics can help further refine the classification of aTNBC patients and provide a basis for individualized treatment.

In the treatment of aTNBC, chemotherapy remains the primary approach due to the lack of well-defined therapeutic targets (29). Treatment regimens containing platinum-based drugs have attracted much attention due to their significant improvement in the prognosis of aTNBC patients. The CBCSG006 study confirmed that for patients with aTNBC, the cisplatin plus gemcitabine regimen in first-line salvage therapy had a longer PFS than the paclitaxel plus gemcitabine regimen (7.73 vs 6.47 months), but it had a higher incidence of adverse reactions, especially in the digestive system and hematological toxicity (30). The results of the tnAcity trial confirmed that, compared with gemcitabine plus carboplatin or gemcitabine plus albumin-bound paclitaxel, the albumin-bound paclitaxel plus carboplatin regimen could significantly prolong the PFS (5.50 vs 6.00 vs 8.30 months) of patients with aTNBC, and there was also an improving trend in OS (12.1 vs 12.6 vs 16.8 months), although the improvement in OS did not reach statistical significance (31). Moreover, The GAP study confirmed that in first-line salvage therapy, the application of albumin-bound paclitaxel combined with cisplatin significantly improved the PFS (9.8 vs 7.4 months, stratified HR= 0.67, 95% CI 0.50-0.88, *P* = 0.088 P=0.004), OS (26.3 vs 22.9 months, stratified HR= 0.62, 95% CI 0.44-0.90, *P*=0.004) and objective response rate (ORR) (81.1% vs. 56.3%, *P* < 0.001) of patients compared with gemcitabine combined with cisplatin (32). Another multicenter real-world study from China demonstrated that, in first-line treatment, platinum-based chemotherapy conferred significant advantages over non-platinum-based regimens in terms of ORR and mPFS (53.0% vs. 32.1%, *P* < 0.001; 8.4 vs. 6.0 months, *P* = 0.022). In contrast, no statistically significant difference in mOS was observed between the two groups (19.2 vs. 16.8 months, *P* = 0.439) (33). In contrast, the PFS (9.63 months [95% CI 8.79 - 9.95]) observed in our study was comparable to that reported in previous studies, while the OS was notably higher. This improvement in OS [27.87 months (95% CI 20.87 - 34.66)] may be attributed to the inclusion of diverse platinum-based treatment regimens in our study. Likewise, the results of the multivariate Cox regression analysis in ours study also indicated that for patients with aTNBC, first-line salvage treatment with platinum-based chemotherapy regimens could reduce the risk of disease progression and death. Further subgroup analysis indicated that patients with dnMBC who received first-line platinum-based therapy had a significantly lower risk of disease progression compared to those with rMBC. Similarly, the risk of death was lower in dnMBC patients treated with first-line platinum-based therapy than in rMBC patients, although this difference did not reach statistical significance. These findings highlight the differences in platinum-based treatment regimens between dnMBC and rMBC, offering new insights for the treatment strategy of aTNBC. However, given the higher toxicity of platinum-based regimens (34), a thorough assessment of the patient’s physical condition is essential when weighing the trade-off between toxicity and survival benefit, particularly for untreated dnMBC patients.

On the other hand, immunotherapy exerts anti-tumor effects by activating or enhancing the patient’s immune system. The IMpassion 130 study demonstrated that atezolizumab, in combination with albumin-bound paclitaxel, significantly improved PFS (7.5 vs 5.0 months, HR=0.62; 95% CI 0.49 - 0.78, *P*<0.001) and OS (25.5 vs 15.0 months, HR=0.62; 95% CI 0.45 - 0.86, *P*<0.001) in PD-L1 positive patients (35). However, the subsequent IMpassion 131 study did not observe the same efficacy in a similar patient population (36). In contrast, the KEYNOTE-355 study showed that pembrolizumab, combined with chemotherapy, could improve PFS (9.70 vs 5.60 months, HR=0.65; 95% CI 0.49 - 0.86, one-sided *P*=0.0012) in aTNBC patients with a PD-L1 expression score (CPS) ≥ 10 (37). Moreover, the Chinese TORCHLIGHT study found that toripalimab, in combination with albumin-bound paclitaxel, significantly improved PFS (8.40 vs 5.60 months, HR=0.65; 95% CI 0.47 - 0.91, *P*=0.010) and OS (32.80 vs 19.50 months, HR=0.62; 95% CI 0.41 - 0.91, *P*=0.015), with a relatively low incidence of adverse reactions (5). Our research results are similar to those of existing key clinical trials, especially in terms of mPFS [8.43 months (95% CI 4.78 - 12.09)]. Moreover, we found that receiving immunotherapy was an independent prognostic factor for improving patient PFS. Similarly, immunotherapy has also shown potential in reducing the risk of death, but this difference has not yet reached statistical significance. Further subgroup analysis did not identify which group—dnMBC or rMBC patients—was more likely to benefit from immunotherapy. However, the findings of the KEYNOTE-355 study suggest that dnMBC patients and rMBC patients with a disease-free interval (DFI) ≥ 12 months may be more likely to achieve better PFS from immunotherapy (37). These results indicate that, although immunotherapy has shown certain efficacy in aTNBC treatment, its effect may vary among different patient subgroups and PD-L1 expression scores. Future studies need to further explore the efficacy of immunotherapy in different aTNBC subgroups and how to optimize treatment regimens to enhance patient survival benefits.

Our study findings indicate that enhanced patient stratification in future trials can boost clinical research precision and effectiveness. By pinpointing distinct patient subgroups and their treatment responses, our research enables more accurate participant selection, homogenizes treatment arms, and mitigates confounding factors. This strategy may yield more reliable trial outcomes and foster the creation of more effective, targeted therapies. However, there are limitations to our study that should be acknowledged. Firstly, the follow-up data collection in this study partially relies on telephone interviews, which may introduce recall bias among participants. This potential bias could subsequently affect the accuracy of recorded survival events and adverse reactions. Secondly, as a single-center retrospective study, it is inherently subject to biases and constrained by a small sample size, which will affect the subgroup analysis efficacy of dnMBC and rMBC, and may also restrict the wide applicability of the research results. Additionally, differences in treatment protocols and clinical practices across various oncology centers could influence patient outcomes, further affecting the applicability of the results. Lastly, while multivariate Cox regression and interaction analyses were used to address potential confounders, unmeasured variables, such as biomarker profiles and socioeconomic status, were not fully considered. Therefore, future studies should include larger sample sizes and real-world data from multiple centers to validate these results, and should also gather data on treatment-related adverse events to provide a more comprehensive understanding of aTNBC management.

## 5 Conclusion

This study indicates that compared with patients with rMBC, patients with dnMBC who undergo first-line treatment have superior survival outcomes. Furthermore, among both dnMBC and rMBC patients, the PFS and OS of first-line treatment exhibit a positive correlation, suggesting that the early treatment response can mirror the long-term prognosis. Hence, differentiating between primary and recurrent metastatic patterns in clinical practice holds significant importance for formulating individualized treatment strategies.

## Data Availability

The raw data supporting the conclusions of this article will be made available by the authors, without undue reservation.
