# Supplementary material for: Analysis of survival differences in advanced triple-negative breast cancer: a real-world study
Source: Front Oncol. 2025 Aug 15;15:1635243. doi: 10.3389/fonc.2025.1635243 (PMC12394513; doi:10.3389/fonc.2025.1635243)
Supplement: Supplementary file 1 [file Table1.doc]

**Table. S1**. Univariate /Multivariate Cox regression analysis and interaction effects for progression-free survival (PFS)

|  | PFS | | | | | | |
| --- | --- | --- | --- | --- | --- | --- | --- |
|  | Univariate Cox regression | | Multivariate Cox regression | | | Interaction Effect | |
| Characteristic | HR (95% CI) | *P* value | HR (95%CI) | | *P* value | HR (95%CI) | *P* value |
| Disease status |  |  |  |  | |  |  |
| rMBC | Ref (1.0) |  | Ref (1.0) |  | | - |  |
| dnMBC | 0.70 (0.51-0.95) | 0.025 | 0.61 (0.43-0.86) | 0.004 | | Ref (1.0) | - |
| Age |  |  |  |  | |  |  |
| <50 | Ref (1.0) |  | Ref (1.0) |  | | - |  |
| ≥50 | 0.95 (0.70-1.29) | 0.753 | 1.06 (0.72-1.56) | 0.781 | | - | - |
| BMI |  |  |  |  | |  |  |
| <18.5 | Ref (1.0) |  | Ref (1.0) |  | | - |  |
| 18.5-24 | 1.81 (1.01-3.27) | 0.058 | 1.84 (0.98-3.47) | 0.059 | | - | - |
| >24 | 1.04 (0.58-1.87) | 0.883 | 1.00 (0.54-1.88) | 0.989 | | - | - |
| Menstrual state |  |  |  |  | |  |  |
| Pre-menopause | Ref (1.0) |  | Ref (1.0) |  | | - |  |
| Post-menopause | 0.87 (0.63-1.21) | 0.389 | 0.87 (0.57-1.35) | 0.545 | | - | - |
| Histological grade |  |  |  |  | |  |  |
| G1 | Ref (1.0) |  | Ref (1.0) |  | | - |  |
| G2 | 1.87 (0.68-5.01) | 0.224 | 1.69 (0.60-4.74) | 0.319 | | - | - |
| G3 | 2.14 (0.78-5.87) | 0.139 | 1.78 (0.62-5.09) | 0.281 | | - | - |
| Pathological classification |  |  |  |  | |  |  |
| Others | Ref (1.0) |  | Ref (1.0) |  | | - | - |
| Invasive ductal carcinoma | 1.28 (0.85-1.90) | 0.234 | 1.01 (0.65-1.57) | 0.972 | | - | - |
| Viscera metastasis |  |  |  |  | |  |  |
| No | Ref (1.0) |  | Ref (1.0) |  | | - |  |
| Yes | 1.16 (0.83-1.63) | 0.372 | 1.06 (0.70-1.60) | 0.796 | | - | - |
| Metastatic sites |  |  |  |  | |  |  |
| 1 | Ref (1.0) |  | Ref (1.0) |  | | - |  |
| ＞1 | 1.04 (0.77-1.39) | 0.799 | 1.01 (0.70-1.46) | 0.945 | | - | - |
| Systemic treatment |  |  |  |  | | - | - |
| Paclitaxel/Anthracycline-based regimen | Ref (1.0) |  | Ref (1.0) |  | | - |  |
| Platinum-based regimen | 0.72 (0.52-0.99) | 0.044 | 0.62 (0.44-0.89) | 0.009 | | 0.77 (0.38-1.55) | 0.465 |
| Immunotherapy | 0.60 (0.40-0.92) | 0.020 | 0.46 (0.29-0.73) | 0.001 | | 1.19 (0.47-3.02) | 0.712 |
| Local treatment of metastasis |  |  |  |  | |  |  |
| No | Ref (1.0) |  | Ref (1.0) |  | | - |  |
| Yes | 0.75 (0.55-1.04) | 0.085 | 0.66 (0.46-0.96) | 0.028 | | 1.07 (0.50-2.32) | 0.854 |

^*^Reference (1.0)

**Table. S2**. Univariate /Multivariate Cox regression analysis and interaction effects for overall survival (OS)

|  | OS | | | | | |
| --- | --- | --- | --- | --- | --- | --- |
|  | Univariate Cox regression | | Multivariate Cox regression | | Interaction Effect | |
| Characteristic | HR (95% CI) | *P* value | HR (95%CI) | *P* value | HR (95%CI) | *P* value |
| Disease status |  |  |  |  |  |  |
| rMBC | Ref (1.0) |  | Ref (1.0) |  | - | - |
| dnMBC | 0.65 (0.45-0.95) | 0.023 | 0.60 (0.41-0.90) | 0.012 | Ref (1.0) | - |
| Age |  |  |  |  |  |  |
| <50 | Ref (1.0) |  | Ref (1.0) |  | - | - |
| ≥50 | 1.04 (0.74-1.46) | 0.807 | 0.79 (0.53-1.18) | 0.259 | - | - |
| BMI |  |  |  |  |  |  |
| <18.5 | Ref (1.0) |  | Ref (1.0) |  | - | - |
| 18.5-24 | 1.33 (0.74-2.48) | 0.368 | 1.60 (0.82-3.13) | 0.166 | - | - |
| >24 | 0.67 (0.37-1.26) | 0.219 | 0.71 (0.36-1.39) | 0.316 | - | - |
| Menstrual state |  |  |  |  |  |  |
| Pre-menopause | Ref (1.0) |  | Ref (1.0) |  | - |  |
| Post-menopause | 1.24 (0.83-1.84) | 0.296 | 1.56 (0.97-2.52) | 0.068 | - | - |
| Histological grade |  |  |  |  |  |  |
| G1 | Ref (1.0) |  | Ref (1.0) |  | - |  |
| G2 | 1.54 (0.51-5.24) | 0.406 | 2.83 (0.82-9.71) | 0.099 | 0.54 (0.07-4.73) | 0.561 |
| G3 | 2.26 (0.93-9.57) | 0.064 | 4.98 (1.46-17.04) | 0.010 | 1.64 (0.21-9.06) | 0.641 |
| Pathological classification |  |  |  |  |  |  |
| Others | Ref (1.0) |  | Ref (1.0) |  | - |  |
| Invasive ductal carcinoma | 0.91 (0.59-1.41) | 0.689 | 0.70 (0.44-1.13) | 0.145 | - | - |
| Viscera metastasis |  |  |  |  |  |  |
| No | Ref (1.0) |  | Ref (1.0) |  | - |  |
| Yes | 1.33 (0.90-1.97) | 0.157 | 1.09 (0.68-1.76) | 0.720 | - | - |
| Metastatic sites |  |  |  |  |  |  |
| 1 | Ref (1.0) |  | Ref (1.0) |  | - |  |
| ＞1 | 1.43 (1.02-2.02) | 0.041 | 1.50 (0.99-2.28) | 0.054 | 1.38 (0.71-2.72) | 0.337 |
| Systemic treatment |  |  |  |  | - |  |
| Paclitaxel/Anthracycline-based regimen | Ref (1.0) |  | Ref (1.0) |  | - |  |
| Platinum-based regimen | 0.61 (0.42-0.89) | 0.011 | 0.45 (0.30-0.68) | 0.001 | 1.04 (0.41-2.62) | 0.937 |
| Immunotherapy | 0.80 (0.50-1.26) | 0.336 | 0.64 (0.39-1.05) | 0.079 | 1.29 (0.47-3.54) | 0.627 |
| Local treatment of metastasis |  |  |  |  |  |  |
| No | Ref (1.0) |  | Ref (1.0) |  | - | - |
| Yes | 0.94 (0.66-1.36) | 0.752 | 0.92 (0.61-1.38) | 0.687 | - | - |

^*^Reference (1.0)

**Fig. S1.** First-line systemic treatment strategies for de novo metastatic breast cancer (dnMBC) and recurrent metastatic breast cancer (rMBC)


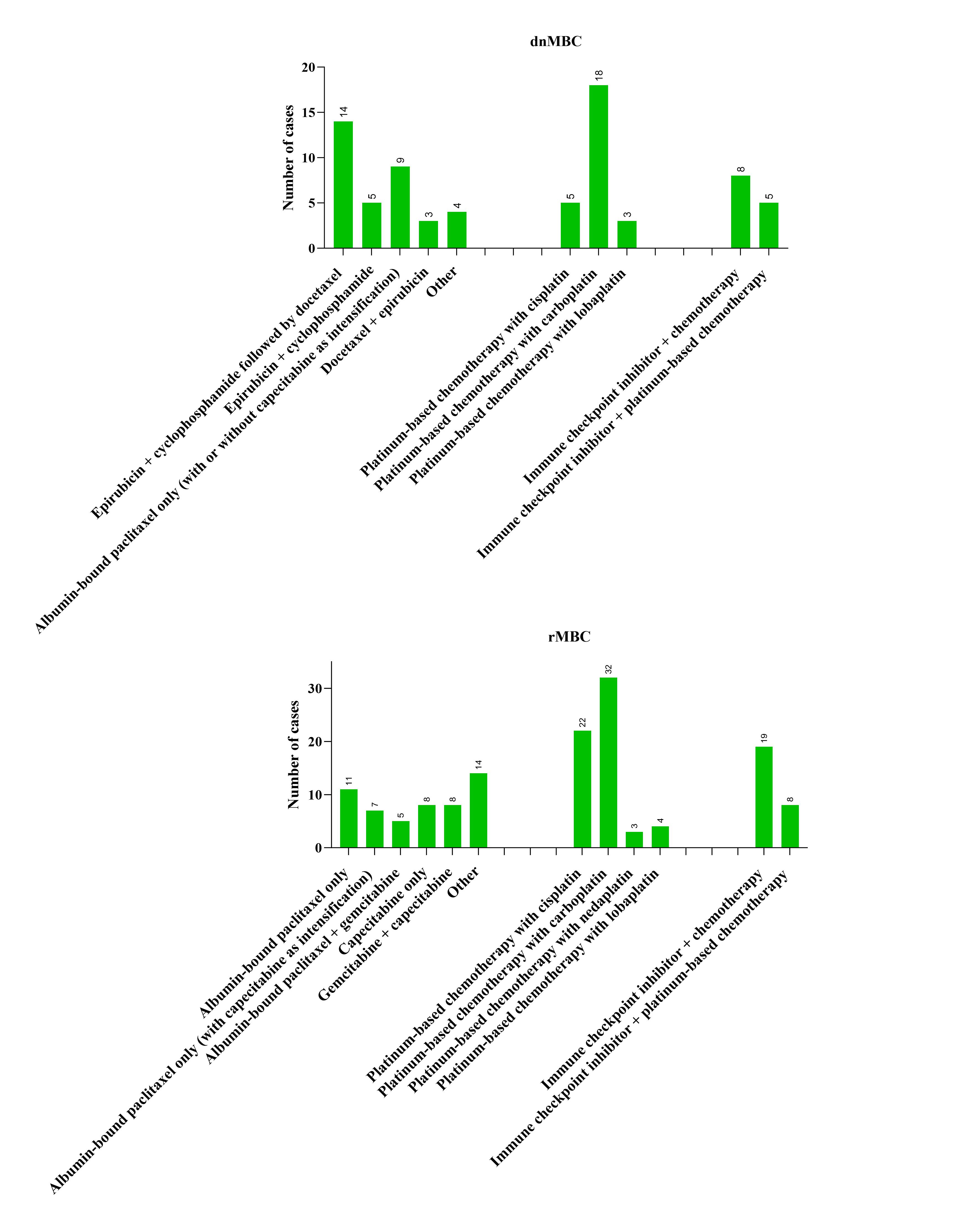


**Fig. S2.** The survival curves of PFS for each treatment subgroup estimated by Kaplan-Meier method


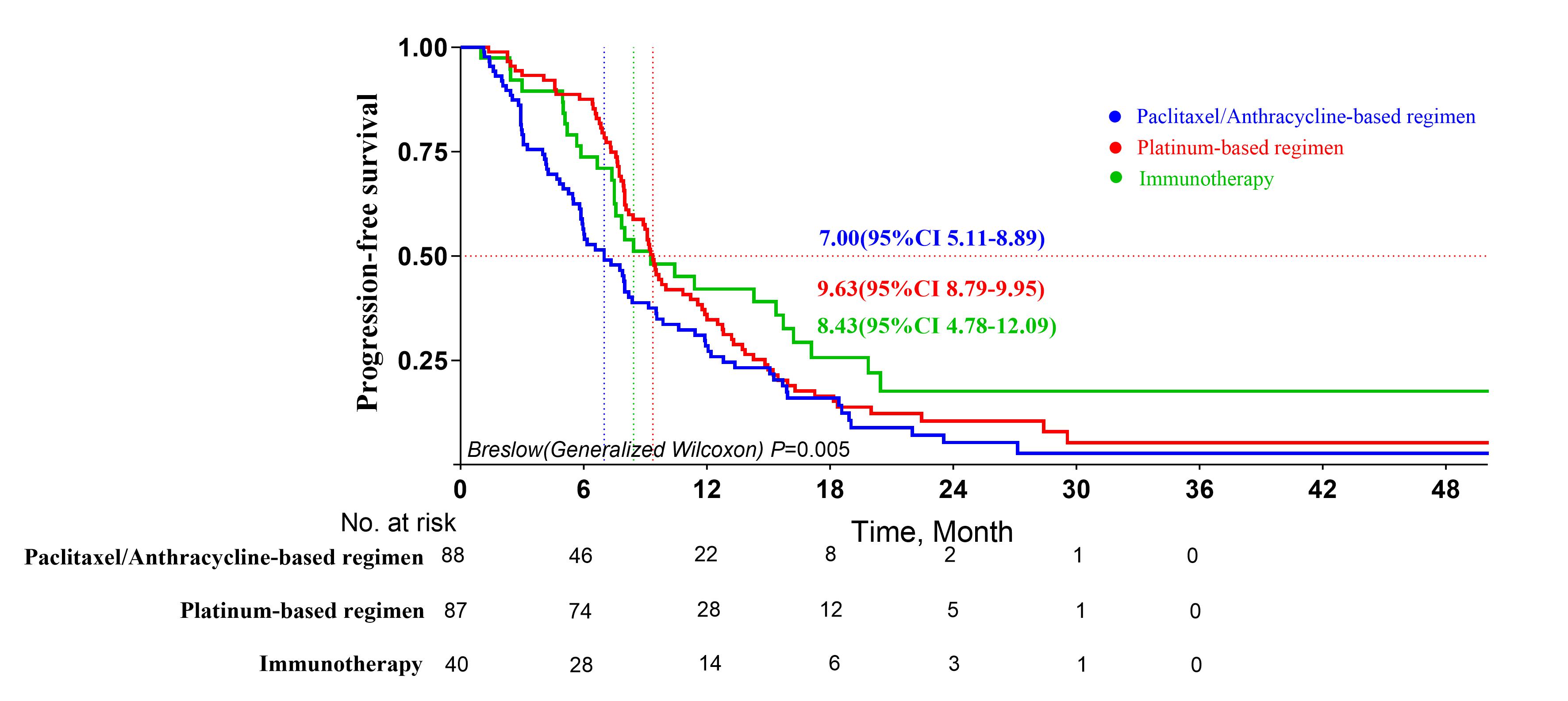


**Fig. S3.** The survival curves of OS for each treatment subgroup estimated by Kaplan-Meier method


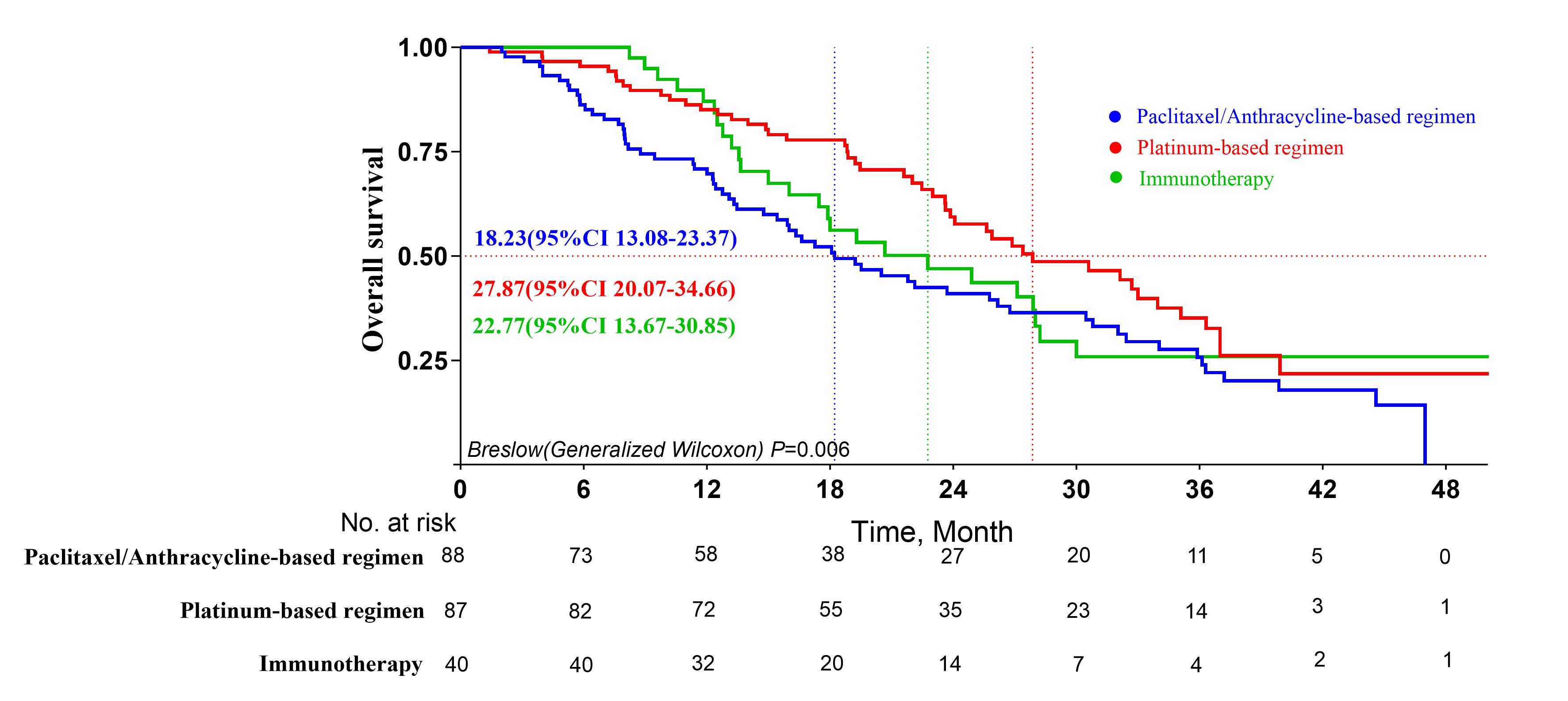


**Table. S3**. Details of the chemotherapy regimens for first-line systemic treatment strategies for dnMBC

| Treatment Regimen | Dose; Administration duration; cycle | number of cases | Cluster analysis |
| --- | --- | --- | --- |
| Epirubicin + cyclophosphamide followed by docetaxel | Epirubicin (90-100mg/m^2^ iv; d1; 21d)  Cyclophosphamide (600mg/m^2^ iv; d1; 21d)  Docetaxel (80-100mg/m^2^ iv; d1; 21d) | 14 | Epirubicin + Cyclophosphamide followed by Docetaxel |
| Epirubicin + cyclophosphamide | Epirubicin (90mg/m^2^ iv; d1;21d) Cyclophosphamide (600mg/m^2^ iv; d1;21d) | 5 | Epirubicin + Cyclophosphamide |
| Albumin-bound paclitaxel only (with capecitabine as intensification) | Albumin-bound Paclitaxel  (260mg/m^2^ iv; d1;21d)  Capecitabine (1000mg/m^2^ bid po; d1-d14;21d) | 4 | Albumin-bound paclitaxel only (with or without capecitabine as intensification) |
| Albumin-bound paclitaxel only (without capecitabine as intensification) | Albumin-bound paclitaxel  (260mg/m^2^ iv; d1;21d) | 5 |
| Docetaxel + epirubicin | Docetaxel (75mg/m^2^ iv; d1; 21d)  Epirubicin (60-90mg/m^2^ iv; d1; 21d) | 3 | Docetaxel + epirubicin |
| Epirubicin + cyclophosphamide followed by docetaxel (with capecitabine as intensification) | Epirubicin (90-100mg/m^2^ iv; d1; 21d)  Cyclophosphamide (600mg/m^2^ iv; d1; 21d)  Docetaxel (80-100mg/m^2^ iv; d1; 21d)  Capecitabine (1000mg/m^2^ bid po; d1-d14;21d) | 2 | Other |
| Albumin-bound paclitaxel + eribulin | Albumin-bound paclitaxel  (100mg/m^2^ iv; d1, d8;21d)  Eribulin (1.4mg/m^2^ iv; d1, d8; d21) | 1 |
| Docetaxel + cyclophosphamide was discontinued due to adverse reactions, and albumin-bound paclitaxel was subsequently administered | Docetaxel (75mg/m^2^ iv; d1; 21d)  Cyclophosphamide (600mg/m^2^ iv; d1; 21d)  Albumin-bound paclitaxel  (260mg/m^2^ iv; d1;21d) | 1 |

**Table. S4**. Details of platinum-based chemotherapy regimens for first-line systemic treatment strategies for dnMBC

| Treatment Regimen | Dose; Administration duration; cycle | number of cases | Cluster analysis |
| --- | --- | --- | --- |
| Cisplatin +vinorelbine | Cisplatin (75mg/m^2^ iv; d1; 21d)  Vinorelbine (25mg/m^2^ iv; d1, d8; 21d) | 2 | Platinum-based chemotherapy with cisplatin |
| Cisplatin + gemcitabine | Cisplatin (25mg/m^2^ iv; d1-d3; 21d)  Gemcitabine (1000mg/m^2^ iv; d1, d8; 21d) | 2 |
| Cisplatin | Cisplatin (80mg/m^2^ iv; d1-d3; 21d) | 1 |
| Albumin-bound paclitaxel + carboplatin | Albumin-bound paclitaxel  (260mg/m^2^ iv; d1;21d)  Carboplatin (AUC=5 iv; d1; d21) | 16 | Platinum-based chemotherapy with carboplatin |
| Albumin-bound paclitaxel + carboplatin followed by vinorelbine | Albumin-bound paclitaxel  (260mg/m^2^ iv; d1;21d)  Carboplatin (AUC=5 iv; d1; 21d)  Vinorelbine (60mg/m^2^ po; d1, d8, d15; 21d) | 1 |
| Epirubicin + cyclophosphamide followed by docetaxel + carboplatin (with capecitabine as intensification) | Epirubicin (90mg/m^2^ iv; d1;21d)  Cyclophosphamide (600mg/m^2^ iv; d1; 21d)  Docetaxel (75mg/m^2^ iv; d1;21d)  Carboplatin (AUC=5 iv; d1; 21d)  Capecitabine (1000mg/m^2^ bid po; d1-d14;21d) | 1 |
| Lobaplatin + vinorelbine | Lobaplatin (30mg/m^2^ iv; d1; 21d)  Vinorelbine (30mg/m^2^ po; d1, d8;21d) | 1 | Platinum-based chemotherapy with lobaplatin |
| Epirubicin + cyclophosphamide followed by docetaxel + lobaplatin | Epirubicin (75mg/m^2^ iv; d1;21d)  Cyclophosphamide (600mg/m^2^ iv; d1; 21d)  Docetaxel (75mg/m^2^ iv; d1;21d)  Lobaplatin (30mg/m^2^ iv; d1; 21d) | 2 |

**Table. S5**. Details of immunotherapy regimens for first-line systemic treatment strategies for dnMBC

| Treatment Regimen | Dose; Administration duration; cycle | number of cases | Cluster analysis |
| --- | --- | --- | --- |
| Sintilimab + albumin-bound paclitaxel | Sintilimab (200mg iv; d1;21d)  Albumin-bound paclitaxel  (260mg/m^2^ iv; d1;21d) | 6 | Immune checkpoint inhibitor + chemotherapy |
| Camrelizumab + albumin-bound paclitaxel | Camrelizumab (200mg iv; d1;21d)  Albumin-bound paclitaxel  (260mg/m^2^ iv; d1;21d) | 2 |
| Camrelizumab combined with albumin-bound paclitaxel + carboplatin | Camrelizumab (200mg iv; d1;21d)  Albumin-bound paclitaxel  (260mg/m^2^ iv; d1;21d)  Carboplatin (AUC=5 iv; d1; 21d) | 2 | Immune checkpoint inhibitor + Platinum-based chemotherapy |
| Sintilimab combined with albumin-bound paclitaxel and carboplatin | Sintilimab (200mg iv; d1;21d)  Albumin-bound paclitaxel  (260mg/m^2^ iv; d1;21d)  Carboplatin (AUC=5-6 iv; d1; 21d) | 3 |

**Table. S6**. Details of the chemotherapy regimens for first-line systemic treatment strategies for rMBC

| Treatment Regimen | Dose; Administration duration; cycle | number of cases | Cluster analysis |
| --- | --- | --- | --- |
| Albumin-bound paclitaxel only | Albumin-bound paclitaxel  (260mg/m^2^ iv; d1;21d) | 11 | Albumin-bound paclitaxel only |
| Albumin-bound paclitaxel only (with capecitabine as intensification) | Albumin-bound paclitaxel  (260mg/m^2^ iv; d1;7d)  Capecitabine (1000mg/m^2^ bid po; d1-d14;21d) | 7 | Albumin-bound paclitaxel only (with capecitabine as intensification) |
| Albumin-bound paclitaxel + gemcitabine | Albumin-bound paclitaxel  (260mg/m^2^ iv; d1, d8;21d)  Gemcitabine (1000mg/m^2^ iv; d1, d8;21d) | 5 | Albumin-bound paclitaxel + gemcitabine |
| Capecitabine only | Capecitabine (1000-1250mg/m^2^ bid po; d1-d14;21d) | 8 | Capecitabine only |
| Gemcitabine + capecitabine | Gemcitabine (1000mg/m^2^ iv; d1, d8;21d)  Capecitabine (1000mg/m^2^ bid po; d1-d14;21d) | 8 | Gemcitabine + capecitabine |
| Albumin-bound paclitaxel + apatinib | Albumin-bound paclitaxel  (260mg/m^2^ iv; d1;21d)  Apatinib (250-500mg/m^2^ qd po; 21d) | 1 | Other |
| Vinorelbine + anlotinib | Vinorelbine (25-30mg/m^2^ iv; d1, d8;21d)  Anlotinib (12mg qd po; d1-d14;21d) | 2 |
| Vinorelbine + temozolomide | Vinorelbine (25-30mg/m^2^ po; d1, d8;21d)  Temozolomide (75-100mg/m^2^ po; d1-d7;21d) | 1 |
| Docetaxel + cyclophosphamide | Docetaxel (75mg/m^2^ iv; d1;21d)  Cyclophosphamide (600mg/m^2^ iv; d1;21d) | 1 |
| Cyclophosphamide only (with capecitabine as intensification) | Cyclophosphamide (50mg/m^2^ po; qd; 21d)  Capecitabine (1250mg/m^2^ bid po; d1-d14;21d) | 1 |
| Albumin-bound paclitaxel + anlotinib | Albumin-bound paclitaxel  (125mg/m^2^ iv; d1, d8;21d)  Anlotinib (12mg qd po; d1-d14;21d) | 2 |
| Pemetrexed only | Pemetrexed (500-600 mg/m^2^ iv; d1;21d) | 1 |
| Epirubicin + cyclophosphamide followed by docetaxel | Epirubicin (90 mg/m^2^ iv; d1;21d)  Cyclophosphamide (600 mg/m^2^ iv; d1;21d)  Docetaxel (100mg/m^2^ iv; d1;21d) | 2 |
| Temozolomide only | Temozolomide (150mg/m^2^ po; d1-d5;21d) | 1 |
| Epirubicin + cyclophosphamide followed by fluorouracil | Epirubicin (90 mg/m^2^ iv; d1;21d)  Cyclophosphamide (600 mg/m^2^ iv; d1;21d)  Fluorouracil (600mg/m^2^ iv; d1;21d) | 1 |
| Epirubicin + vinorelbine | Epirubicin (90 mg/m^2^ iv; d1;21d)  Vinorelbine (25mg/m^2^ iv; d1, d8; 21d) | 1 |

**Table. S7**. Details of platinum-based chemotherapy regimens for first-line systemic treatment strategies for rMBC

| Treatment Regimen | Dose; Administration duration; cycle | number of cases | Cluster analysis |
| --- | --- | --- | --- |
| Cisplatin + albumin-bound paclitaxel | Cisplatin (75-80mg/m^2^ iv; d1; 21d)  Albumin-bound paclitaxel (125-150mg/m^2^ iv; d1;21d) | 4 | Platinum-based chemotherapy with cisplatin |
| Cisplatin + gemcitabine | Cisplatin (75-80mg/m^2^ iv; d1; 21d)  Gemcitabine (1000-1250mg/m^2^ iv; d1, d8; 21d) | 14 |
| Cisplatin + vinorelbine | Cisplatin (75mg/m^2^ iv; d1; 21d)  Vinorelbine (25-30mg/m^2^ iv; d1, d8; 21d) | 4 |
| Carboplatin + eribulin | Carboplatin (AUC=4 iv; d1; d21)  Eribulin (1.4mg/m^2^ iv; d1, d8; d21) | 1 | Platinum-based chemotherapy with carboplatin |
| Carboplatin + vinorelbine | Carboplatin (AUC=4 iv; d1; d21)  Vinorelbine (25-30mg/m^2^ iv; d1, d8; 21d) | 6 |
| Albumin-bound paclitaxel + carboplatin | Albumin-bound paclitaxel (100-150mg/m^2^ iv; d1; d21)  Carboplatin (AUC=4-6 iv; d1; d21) | 18 |
| Gemcitabine + carboplatin | Gemcitabine (1000-1250mg/m^2^ iv; d1; d21)  Carboplatin (AUC=4-5 iv; d1; d21) | 5 |
| Albumin-bound paclitaxel + carboplatin (with capecitabine as intensification) | Albumin-bound paclitaxel (100mg/m^2^ iv; d1; d21)  Carboplatin (AUC=5 iv; d1; d21)  Capecitabine (1250mg/m^2^ bid po; d1-d14;21d) | 1 |
| Epirubicin + cyclophosphamide followed by docetaxel + carboplatin | Epirubicin (90mg/m^2^ iv; d1; d21)  Cyclophosphamide (600mg/m^2^ iv; d1; d21)  Docetaxel (75mg/m^2^ iv; d1; d21)  Carboplatin (AUC=5 iv; d1; d21) | 1 |
| Nedaplatin + pemetrexed + bevacizumab | Nedaplatin (90mg/m^2^ iv; d1; d21)  Pemetrexed (500mg/m^2^ iv; d1; d21)  Bevacizumab (15mg/kg iv; d1; d21) | 1 | Platinum-based chemotherapy with nedaplatin |
| Nedaplatin + eribulin | Nedaplatin (80-100mg/m^2^ iv; d1; d21)  Eribulin (1.4mg/m^2^ iv; d1, d8; d21) | 2 |
| Albumin-bound paclitaxel + lobaplatin (with capecitabine as intensification) | Albumin-bound paclitaxel (260mg/m^2^ iv; d1; 21d)  Lobaplatin (30mg/m^2^ iv; d1; 21d)  Capecitabine (1000mg/m^2^ bid po; d1-d14;21d) | 1 | Platinum-based chemotherapy with lobaplatin |
| Albumin-bound paclitaxel + lobaplatin | lbumin-bound paclitaxel (260mg/m^2^ iv; d1; 21d)  Lobaplatin (40mg/m^2^ iv; d1; 21d) | 1 |
| Gemcitabine + lobaplatin | Gemcitabine (1000mg/m^2^ iv; d1, d8; d21)  Lobaplatin (40mg/m^2^ iv; d1; 21d) | 1 |
| Docetaxel + lobaplatin | Docetaxel (75mg/m^2^ iv; d1; d21)  Lobaplatin (40mg/m^2^ iv; d1; 21d) | 1 |

**Table. S8**. Details of immunotherapy regimens for first-line systemic treatment strategies for rMBC

| Treatment Regimen | Dose; Administration duration; cycle | number of cases | Cluster analysis |
| --- | --- | --- | --- |
| Sintilimab + anlotinib combined with vinorelbine or capecitabine | Sintilimab (200mg iv; d1;21d)  Anlotinib (12mg qd po; d1-d14;21d)  Vinorelbine (25-30mg/m^2^; d1, d8;21d)  Capecitabine (1000mg/m^2^ bid po; d1-d14;21d) | 8 | Immune checkpoint inhibitor + chemotherapy |
| Sintilimab + albumin-bound paclitaxel | Sintilimab (200mg iv; d1;21d)  Albumin-bound paclitaxel  (260mg/m^2^ iv; d1;21d) | 5 |
| Pembrolizumab + albumin-bound paclitaxel | Pembrolizumab (200mg iv; d1;21d)  Albumin-bound paclitaxel  (260mg/m^2^ iv; d1;21d) | 4 |
| Atezolizumab + albumin-bound paclitaxel | Atezolizumab (840mg iv; d1, d15;28d)  Albumin-bound paclitaxel  (100mg/m^2^ iv; d1, d8, d15;28d) | 1 |
| Tislelizumab + eribulin + bevacizumab | Tislelizumab (200mg iv; d1;21d)  Eribulin (1.4mg/m iv; d1, d8;21d)  Bevacizumab (15mg/kg; d1;21d) | 1 |
| Sintilimab combined with albumin-bound paclitaxel + cisplatin | Sintilimab (200mg iv; d1;21d)  Albumin-bound paclitaxel  (260mg/m^2^ iv; d1;21d)  Cisplatin (75mg/m^2^ iv; d1; 21d) | 1 | Immune checkpoint inhibitor + Platinum-based chemotherapy |
| Sintilimab combined with gemcitabine + cisplatin | Sintilimab (200mg iv; d1;21d)  Gemcitabine (1000mg/m^2^ iv; d1, d8; d21)  Cisplatin (25mg/m^2^ iv; d1-d3; 21d) | 1 |
| Camrelizumab combined with gemcitabine + cisplatin | Camrelizumab (200mg iv; d1;21d)  Gemcitabine (1000mg/m^2^ iv; d1, d8; d21)  Cisplatin (25mg/m^2^ iv; d1-d3; 21d) | 1 |
| Camrelizumab combined with albumin-bound paclitaxel and carboplatin | Camrelizumab (200mg iv; d1;21d)  Albumin-bound paclitaxel  (260mg/m^2^ iv; d1;21d)  Carboplatin (AUC=5 iv; d1; 21d) | 3 |
| Sintilimab combined with albumin-bound paclitaxel and carboplatin | Sintilimab (200mg iv; d1;21d)  Albumin-bound paclitaxel  (125mg/m^2^ iv; d1, d8;21d)  Carboplatin (AUC=5 iv; d1; 21d) | 1 |
| Trilaciclib combined with albumin-bound paclitaxel and carboplatin | Trilaciclib (240mg iv; d1;21d)  Albumin-bound paclitaxel  (26mg/m^2^ iv; d121d)  Carboplatin (AUC=5 iv; d1; 21d) | 1 |

**Table. S9**. Treatment-related adverse effects (AEs).

| **Event** | **Patients, (%)** | | |
| --- | --- | --- | --- |
|  | **Any grade (n/%)** | **G1-2 (n/%)** | **G3-4 (n/%)** |
| Anemia | 156 (72.5%) | 141 (65.6%) | 15 (7.0%) |
| Neutropenia | 169 (78.6%) | 133 (61.9%) | 36 (16.7%) |
| Lymphocytopenia | 10 (4.6%) | 10 (4.7%) | 0 (0%) |
| Thrombocytopenia | 23 (10.7%) | 23 (10.7%) | 0 (0%) |
| Peripheral neuropathy | 69 (32.1%) | 67 (31.2%) | 2 (0.9%) |
| Diarrhea | 25 (11.6%) | 17 (8.0%) | 1 (0.4%) |
| Anorexia | 29 (13.4%) | 29 (13.5%) | 0 (0%) |
| Fatigue | 20 (9.3%) | 20 (9.3%) | 0 (0%) |
| Rash | 3 (1.3%) | 1 (0.4%) | 0 (0%) |
| Blurred vision | 1 (0.4%) | 1 (0.4%) | 0 (0%) |
| Transaminase increasing | 49 (22.8%) | 39 (18.1%) | 10 (4.6%) |
| Nausea or vomiting | 150 (69.8%) | 137 (63.7%) | 13 (6.0%) |
| Edema | 1 (0.4%) | 1 (0.4%) | 0 (0%) |
| Myalgia | 14 (6.5%) | 14 (6.5%) | 1 (0.4%) |
| Stomatitis | 2 (0.9%) | 1 (0.4%) | 1 (0.4%) |
| Thyroiditis | 1 (0.4%) | 1 (0.4%) | 0 (0%) |
| Pneumonia | 1 (0.4%) | 1 (0.4%) | 0 (0%) |

There were no grade 5 drug-related AEs.
